# Local weather affects body condition of three North American songbird species on the Texas Coast

**DOI:** 10.1002/ece3.10317

**Published:** 2023-07-15

**Authors:** Michael W. D. McCloy, Selma Glasscock, Jacquelyn K. Grace

**Affiliations:** ^1^ Texas A&M University College Station Texas USA; ^2^ Welder Wildlife Foundation Sinton Texas USA

**Keywords:** avian ecology, climate change, conservation, hurricane, physiology, resilience

## Abstract

Body condition is a frequently used physiological indicator of avian health and can be affected by an array of environmental variables. Although a number of studies have investigated the specific effects of individual weather variables on body condition in birds, few have analyzed the effects of both temperature and precipitation within the context of an extreme weather event such as hurricanes. In this study, we examined the relationship between breeding‐season body condition and daily maximum temperature, daily minimum temperature, and monthly total precipitation for three passerine bird species at the Welder Wildlife Refuge near Rockport, Texas. We also evaluated yearly changes in body condition over a 12‐year period for northern cardinals (*Cardinalis cardinalis*), painted buntings (*Passerina ciris*), and white‐eyed vireos (*Vireo griseus*), focusing on the extreme precipitation event of Hurricane Harvey, which caused heavy localized flooding. We found that body condition declined with average daily minimum and maximum temperatures, while precipitation had varied, species‐specific effects in the three species analyzed. Our results also suggest that northern cardinals experienced a notable reduction in average body condition in the 2 years following Hurricane Harvey. Taken together, we conclude that short‐term precipitation and temperature drivers can be important correlates of body condition in songbirds and that severe weather events may reduce body condition in some bird species.

## Graphical abstract

This study demonstrates that the body condition of three songbird species, as quantified by scaled mass index, can be affected by short‐term changes in precipitation and temperature. The continued and expected future effects of climate change render these findings especially relevant and highlight the need for further related research.

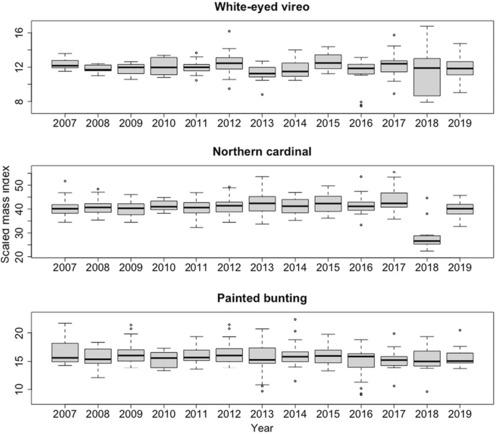

## 1 INTRODUCTION

Coastal ecosystems are particularly vulnerable to impacts by natural disasters such as hurricanes, of which the frequency and intensity are rising (Smith et al., 2010) and are expected to further increase in the future (Bender et al., 2010). To plan for the recovery, management, and conservation of these ecosystems we must first understand the ecological dynamics of postdisturbance recovery. The response of bird communities to natural disasters is of particular interest, in part because avian diversity can be used as a functional indicator for entire ecosystem health (Bregman et al., 2016; Smits & Fernie, 2013) and birds are valuable providers of ecosystem services (Sekercioglu, 2006). As bird populations decline, negative effects on these ecosystem services and their resulting value to human societies may occur (Sekercioglu et al., 2004).

Responses to hurricanes and other natural disasters can be studied in birds in numerous ways (Coppedge et al., 2008; McCloy et al., 2022; Sigel et al., 2006), including through species diversity (Blair, 1996), community assemblages (Crooks et al., 2004), population dynamics (SÆther et al., 2004), and individual physiology (Schoech et al., 2011). Body size along with body mass adjusted for size (i.e., body condition) are two physiological measures that are easily measured in the field by practitioners and may provide insight into sublethal effects of disturbance on individuals and populations. Body condition is a useful indicator of disease prevalence, individual health, and fitness in vertebrates including birds (Granthon & Williams, 2017; Stevenson & Woods, 2006), bison (Vervaecke et al., 2005), and some reptiles (Romero et al., 2001). Body size can reflect early‐life stressor experiences (Grace et al., 2017; Schoech et al., 2011). In songbirds, both measures are responsive to climate change and acute disturbance events (McLean et al., 2018). For example, climate change is correlated with a decrease in body condition, fat scores, and longer average wing lengths in several species (Collins et al., 2017; Van Buskirk et al., 2010).

Hurricanes are often accompanied by sudden extreme increases in precipitation, which are expected to become more intense, globally, with continued climate change (O'Gorman, 2015). In migratory songbirds, the links between winter rainfall and/or habitat moisture, food availability, and body condition are well established (e.g., Akresh et al., 2019a, 2019b; Angelier et al., 2011; McKinnon et al., 2015; Studds & Marra, 2007). In general, decreased precipitation on the wintering grounds drives decreases in food availability, which then leads to decreased avian body condition (e.g., McKinnon et al., 2015, Smith et al., 2010, Studds & Marra, 2007) that can carry over to impact reproductive success during the breeding season (Rockwell et al., 2017). Relationships between body condition and precipitation on the breeding grounds are also evident in the literature. For some species, increased precipitation is positively correlated with body condition until a threshold is reached, after which body condition declines (e.g., male great snipe, *Gallinago media*, Witowska et al., 2022). For others, precipitation during the breeding season is negatively correlated with fitness measures including reproductive success, juvenile survival, and parental survival (e.g., northern wheatear, *Oenanthe oenanthe*, Öberg et al., 2015, grassland birds, Zuckerberg et al., 2018, wire‐tailed manakin, *Pipra filicauda*, Ryder and Sillett 2006), due in part to declines in parental nest visitation rates (Öberg et al., 2015) along with flooding and patch‐specific variation in microclimates (Zuckerberg et al., 2018). Shifts in larger‐scale precipitation patterns are also linked to variation in natural selection on birds (Siepielski et al., 2017), indicating that precipitation changes have the potential for long‐term impacts on populations.

In addition to precipitation, other mechanisms can influence the effects of hurricanes on birds. For example, high wind speeds in conjunction with extreme precipitation may cause direct mortality of birds (Michener et al., 1997), and destruction of avian habitat and food resources (Wiley & Wunderle, 1993). These effects are typically strongest within the year after a hurricane, although population dynamics may be altered for years afterward (Michener et al., 1997). The strength of indirect effects on avifauna are mediated by numerous species‐specific and ecological factors, including the specificity of vegetative reliance, foraging guild, endemic status, and seasonal timing of the hurricane. Species that rely on specific vegetation structures for foraging, hunting, or nesting often experience short‐term decreases in population size following a hurricane due to limited food availability and reduced cover while species with broader vegetative relationships are largely unaffected (Wiley & Wunderle, 1993). Species with granivorous, nectivorous, and frugivorous dietary preferences may be more negatively affected by hurricanes than insectivorous species, because of the quick posthurricane recovery time of many insect populations, coupled with the high level of dietary specialization for many granivores, nectivores, and frugivores (Ackerman et al., 1991; Askins & Ewert, 1991). Finally, breeding‐season disturbances oftentimes have the greatest long‐term effects on populations due to the vulnerability of juvenile and adult populations at these times (Michener et al., 1997).

In this study, we sought to elucidate how the body condition of three passerine bird species near Rockport, Texas responded to changes in temperature and precipitation, particularly in the three breeding seasons following the extreme precipitation event of Hurricane Harvey. Hurricane Harvey made initial landfall on August 25, 2017, as a Category 4 hurricane just east of Rockport on the northern end of San Jose Island. The hurricane then made a second landfall on the northeast side of Copano Bay several hours later. Hurricane Harvey had the highest storm‐total precipitation ever recorded in the United States and its economic cost was second to only Hurricane Katrina (Blake & Zelinsky, 2018). The impact of Hurricane Harvey in the immediate vicinity of our study location provided a unique opportunity to analyze site‐specific shifts in avian body condition in subsequent years.

We generated a series of hypotheses concerning the effects of age and various weather variables, predicting a differential response across species. First, we hypothesized (H1) precipitation would have a positive effect on scaled mass index of northern cardinal (*Cardinalis cardinalis*; resident) and painted bunting (*Passerina ciris*, migrant), only up to a certain threshold. These species maintain at least a partially granivorous diet (Billerman et al., 2022) and low precipitation totals may decrease seed production, while an overabundance of precipitation may cause flooding and destroy seed‐producing vegetation (Oram et al., 2021). We also predicted that (H2) minimum temperature would have a positive effect and maximum temperature would have a threshold effect on the scaled mass index of these two species, as seed production may be affected by extreme temperatures. Lastly, we hypothesized (H3) that the scaled mass index of white‐eyed vireo (*Vireo griseus*, migrant), which is largely insectivorous (Hopp, 2022), will be positively affected by precipitation and minimum temperature up to a certain threshold, but negatively affected by maximum temperature because insects are highly sensitive to changes in temperature and precipitation (Ma et al., 2021; Møller, 2019; Rebaudo et al., 2016). Additionally, migrant species such as the white‐eyed vireo and painted bunting may demonstrate higher variation in body condition due in part to spillover effects from conditions on the wintering grounds (Akresh et al., 2019a; Rockwell et al., 2017).

## 2 MATERIALS AND METHODS

We analyzed 13 years of data from the Monitoring Avian Productivity and Survivorship (MAPS) banding station at the Welder Wildlife Refuge, using scaled mass index to identify changes in body condition among three avian species in relation to year, precipitation, and temperature. Scaled mass index is a condition index calculated using both body mass and morphometric measurements (i.e., wing length) to assess overall body condition (Peig & Green, 2009). Condition indices have long been used in studies of avian physiology, and the use of multivariate measures is recognized as the most accurate means of doing so (Freeman & Jackson, 1990). A variety of methods exist for quantifying avian body condition through condition indices, and there is still ongoing debate as to which method is generally the “best” (Akresh et al., 2019a; Labocha et al., 2014). Here we chose to use scaled mass index (Peig & Green, 2009), which is an effective and well‐regarded measure of body condition in birds (Danner et al., 2013; English et al., 2018; Peig & Green, 2009). Scaled mass index has previously been shown to perform better than numerous other condition indices because it uses a multiplicative error function, which better accounts for the scaling between mass and body length in an individual over time (Danner et al., 2013; Peig & Green, 2010).

### 2.1 Study area

**FIGURE 1:**
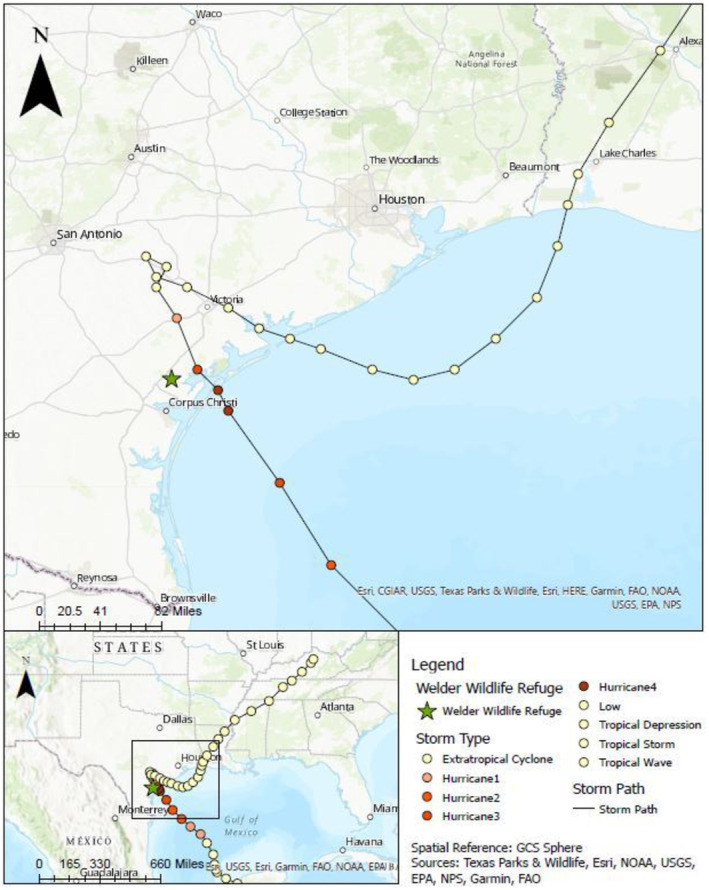
Map of the location of Welder Wildlife Refuge within the state of Texas, with inset map showing track of Hurricane Harvey, and its progression from a hurricane to progressively weaker storm types, including tropical cyclone, and tropical depression.

The Welder Wildlife Refuge is a 7800‐acre tract of privately owned land in San Patricio County, Texas about 64 km north of Corpus Christi and within the greater Texas Coastal Bend ecosystem (Figure 1). The second landfall of Hurricane Harvey was approximately 21 km from the eastern boundary of the Welder Wildlife Refuge (Figure 1). We specifically analyzed data from the Mesquite Pasture (MEPA) MAPS station, which is located near the center of the southern boundary of the refuge. Multiple MAPS station locations have been operated at the Welder Wildlife Refuge over the years, and we exclusively used data from the MEPA location because (1) it was operated continuously from 2007 to 2019, and (2) to avoid additional bias caused by incorporating multiple capture locations into this analysis.

### 2.2 Data collection

Banding operations were carried out between the years 2007 and 2019 following standardized MAPS protocol established by the Institute for Bird Populations. The complete and current version of the MAPS protocol is described in DeSante et al. (2015), with an abbreviated description provided, here. Between 10 and 11 mist nets were operated for 1 day in each 10‐day period during the breeding season (May to August) for up to 6 h following sunrise (i.e., highest bird activity) (Robbins, 1981). At the Welder Wildlife Refuge, mist nets were typically operated for only 3–4 h postsunrise due to rapidly rising temperatures in conjunction with very high humidity levels. Once temperatures reached 90 degrees Fahrenheit, the nets were closed for the day to ensure the safety of any birds captured. Each mist net was 12 m long and 2.5 m tall with a 30 mm mesh. Nets were checked every 20–30 min throughout the period in which they were open. All birds captured were identified to species, sex, and age based on Pyle (1997) except for unidentified migrant *Empidonax* genus flycatchers. Birds were banded with a nine‐digit aluminum band from the U.S. Geological Survey, and wing and mass were measured. Data were recorded in the field on paper datasheets and subsequently entered into MAPSPROG, the dedicated database for storing all MAPS data from stations across the US, where they were proofed and checked for quality. Data were then formatted in Microsoft Excel and statistical analyses were performed in R (v 4.2.1; R Core Team, 2021).

We downloaded weather data from DayMet (Thornton et al., 2021) using the Single Pixel Extraction Tool. This provided a 1 km^2^ resolution of surface weather data from a given set of coordinates. Then, we analyzed total precipitation, average maximum temperature, and average minimum temperature for the 30 days prior to the capture of each individual bird. For example, if an individual bird was captured on 15 May, weather data for that bird was collected for the period between 16 April and 15 May. Hurricane Harvey made landfall in August 2017, which fell outside the temporal window of the MAPS protocol. Thus, avian data was not collected in the 30 days immediately following the hurricane impact and subsequent data was collected beginning in May 2018. We chose to analyze these temperature and precipitation variables for two main reasons: first, these data were readily available on a fine spatiotemporal scale. Second, the existing literature allowed us to make a priori predictions (Gardner et al., 2018) about their effects on avian body condition. Despite their availability in DayMet, we did not include additional weather variables such as wind, relative humidity, and solar radiation to minimize the risk of overfitting our models, and because we predicted a priori that these variables would not have notable effects on avian body condition.

Effects of weather on animals can vary temporally (English et al., 2018; Salewski et al., 2013), and therefore, we carefully considered the time frame of weather variables we selected. We chose to use a 30‐day time frame with the intent to capture short‐term effects of extreme weather (such as dehydration due to high heat) alongside effects due to longer‐term changes in resource availability.

### 2.3 Scaled mass index calculation

We calculated scaled mass index for each species using the following equation:

$${\hat{M}}_{i}=M_{i}{\left[\frac{L_{0}}{L_{i}}\right]}^{\left(b_{\mathrm{SMA}}\right)}$$

where ${\hat{M}}_{i}$ is scaled mass index value, *M*_*i*_ is body mass, *L*_0_ is average population wing length calculated across all years, *L*_*i*_ is wing length, and *b*_SMA_ is the scaling exponent, calculated as the slope of the Standardized Major Axis (SMA) regression line across ln‐transformed data of *M*_*i*_ and *L*_*i*_ (Peig & Green, 2009). This was calculated in JMP Pro (v. 14.0.0) using a bivariate fit of the ln of mass to the ln of wing length and then calculating an orthogonal fit ratio (SMA regression line). We calculated scaled mass index values across sex for each species in this study because the relationship between size and mass did not differ significantly between sexes.

### 2.4 Statistical analyses

We analyzed the scaled mass index of the three most frequently captured species at the Welder MAPS station between 2007 and 2019: northern cardinal, painted bunting, and white‐eyed vireo. Individuals without either wing length and/or body mass data were excluded from analyses since both values are needed to calculate scaled mass index values. Recaptured individuals were excluded from analysis to prevent potential bias caused by repeated captures of the same individual bird.

**FIGURE 2:**
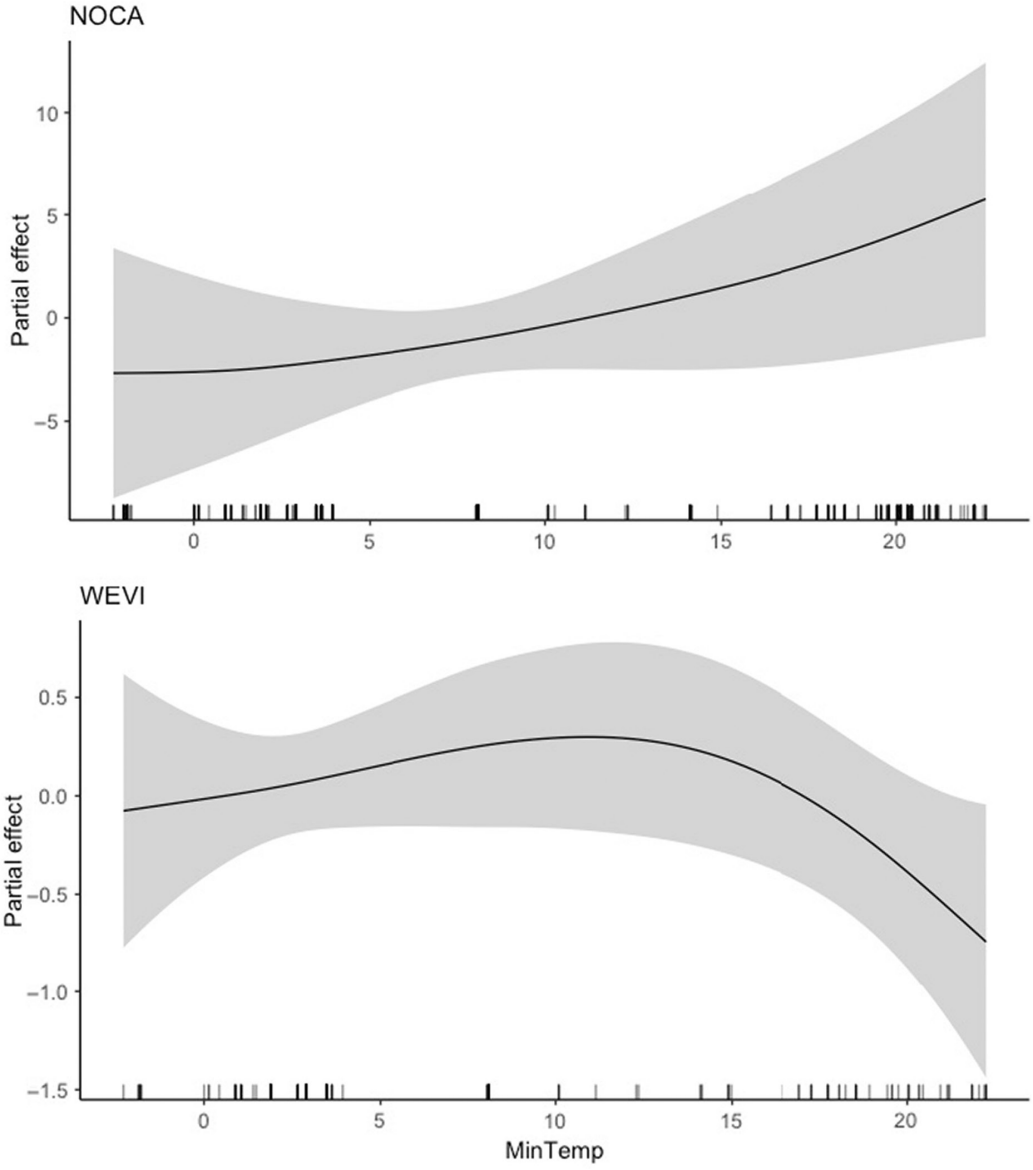
Partial effect of average daily minimum temperature on scaled mass index for northern cardinal (NOCA, top) and white‐eyed vireo (WEVI, bottom), conditional upon the other estimated terms in the model. Black lines on *x*‐axis represent data point distribution. Values on the *y*‐axis indicate where the effect of average daily minimum temperature reduces scaled mass index below (negative values) and above (positive values) the overall mean scaled mass index (zero).

**FIGURE 3:**
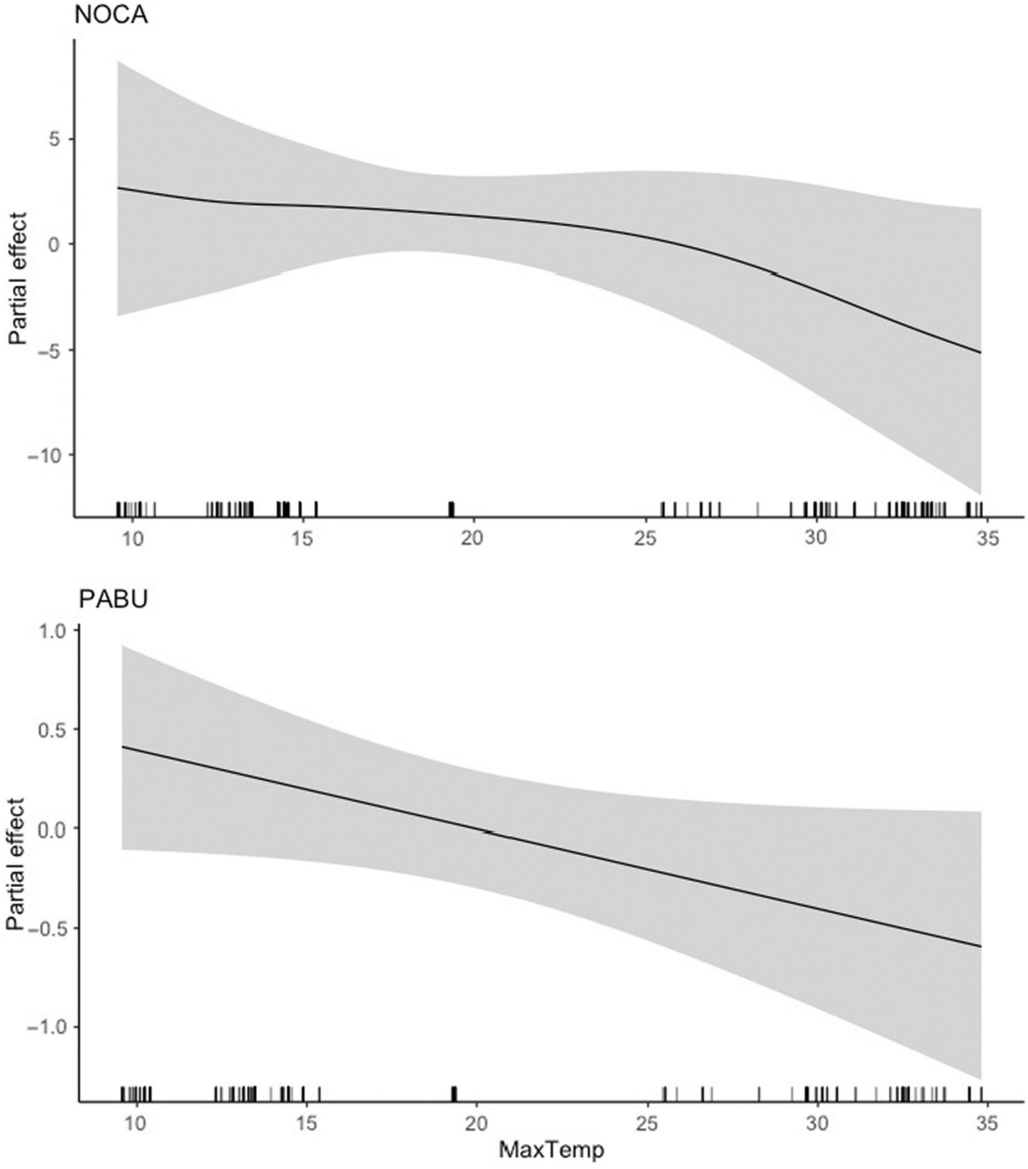
Partial effect of average daily maximum temperature on scaled mass index for northern cardinal (NOCA, top) and painted bunting (PABU, bottom). Values on the *y*‐axis indicate where the effect of average daily minimum temperature reduces scaled mass index below (negative values) and above (positive values) the overall mean scaled mass index (zero).

**FIGURE 4:**
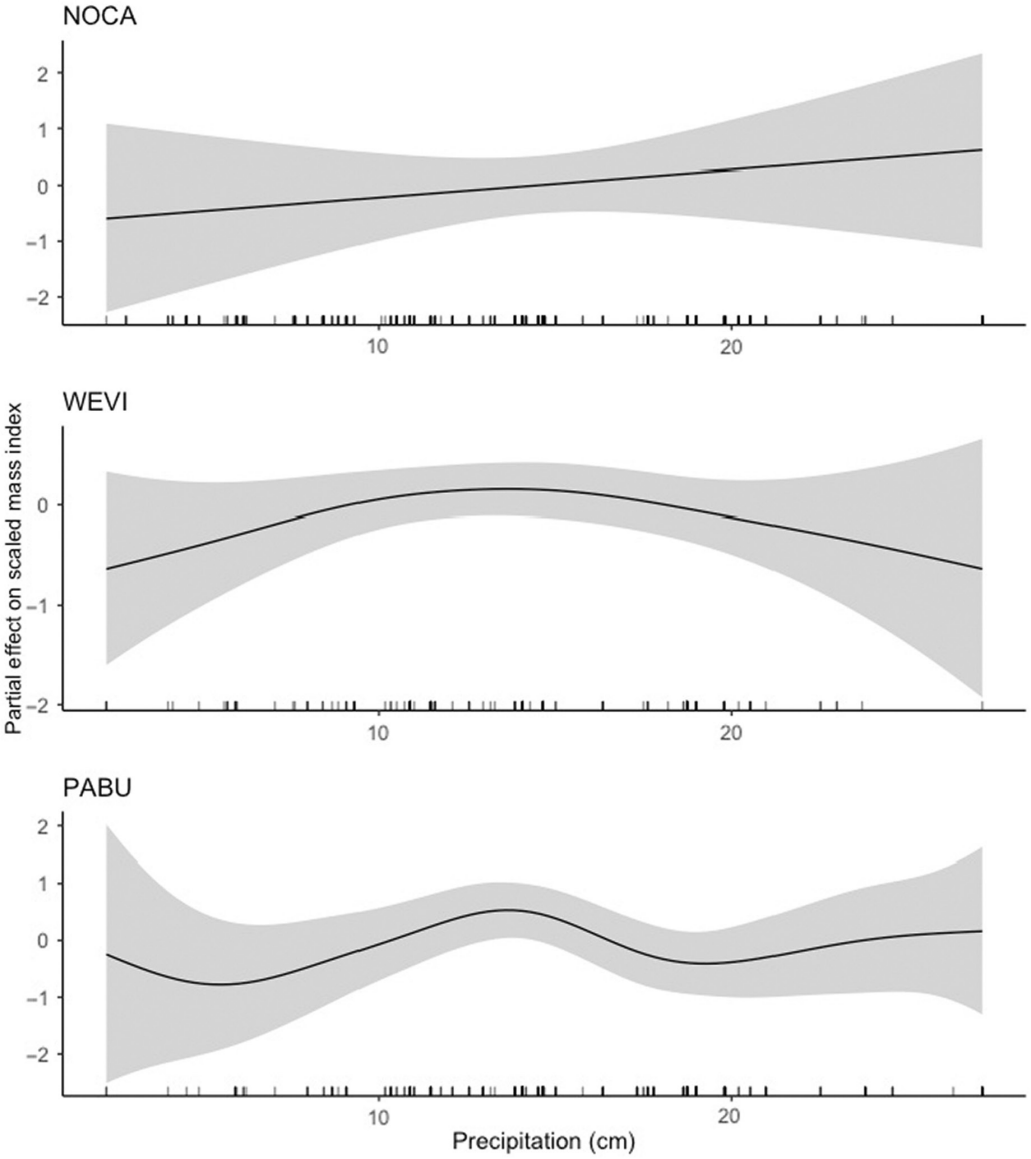
Partial effect of total precipitation on scaled mass index for northern cardinal (NOCA, top), painted bunting (PABU, center) and white‐eyed vireo (WEVI, bottom). Values on the *y*‐axis indicate where the effect of average daily minimum temperature reduces scaled mass index below (negative values) and above (positive values) the overall mean scaled mass index (zero).

**FIGURE 5:**
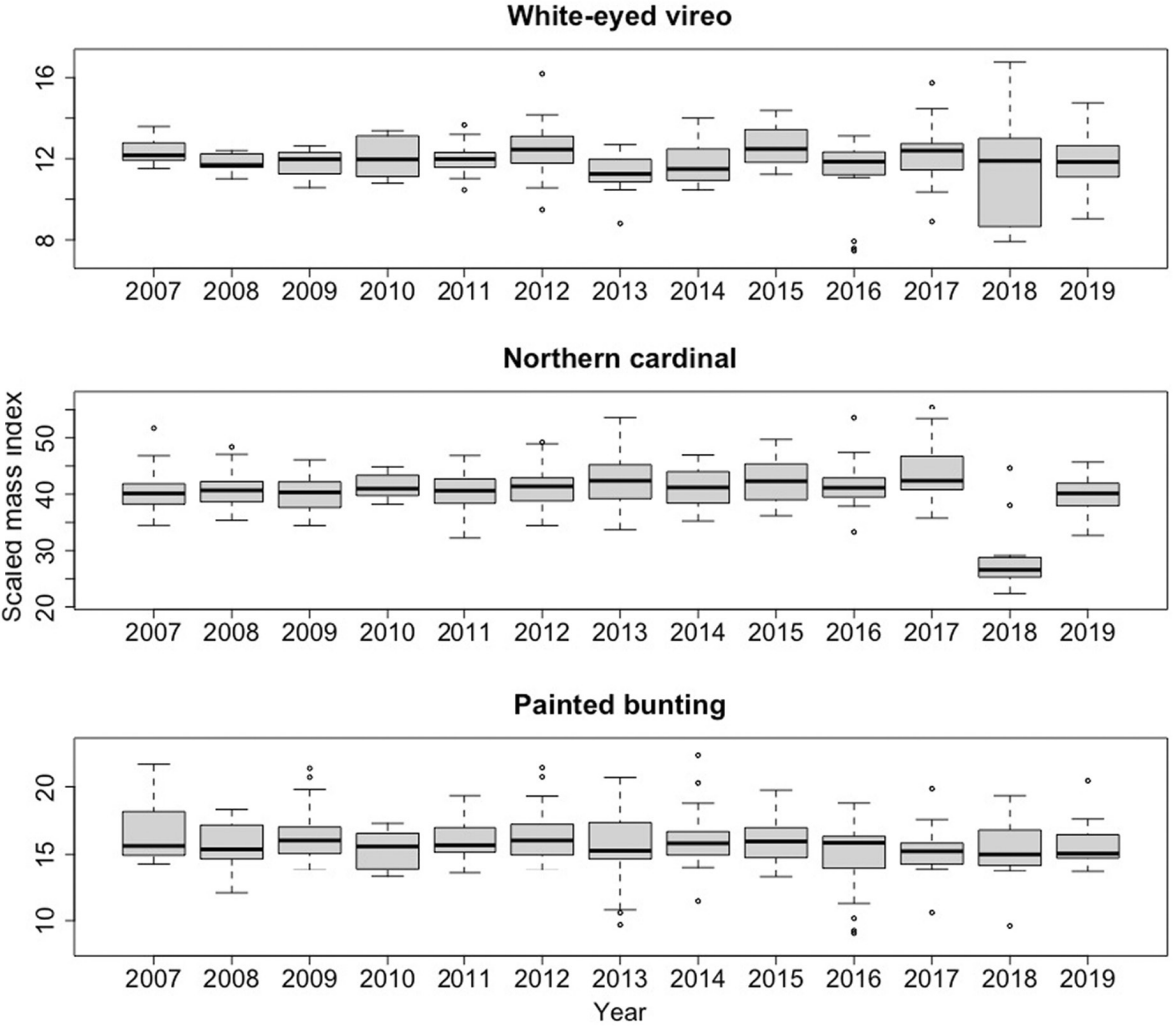
Boxplot of scaled mass index by year from 2007 to 2019 for white‐eyed vireo, northern cardinal, and painted bunting.

We constructed a series of nonparametric generalized additive models (GAMs; Gaussian, identify link function) with the “mgcv” package (Wood, 2011) in the R statistical programming language (v 4.2.1; R Core Team, 2021) to evaluate the effects of selected variables on avian body condition. Unlike generalized linear models or linear mixed effects models, GAMs do not make a priori assumptions about the relationship between variables. Their effects are additive, and smooths are fitted with smoothing splines by cubic polynomials. As a result, GAMs are a popular ecological modeling technique for large datasets and/or for multiple regression functions (Wood et al., 2014; Yee & Mitchell, 1991). We estimated the degree of smoothness of model terms through a maximum‐likelihood approach, which allows each term to be penalized to zero (Viana & Chase, 2022). Our global models included the following smooth terms: average daily minimum temperature, average daily maximum temperature, total precipitation, year (2007–2019), sex, age (hatch year or after hatch year, subsequently referred to as “juvenile” or “adult”), and the random effect of date. Age, sex, and year were included as linear effects. *Y*‐axis values in partial effect plots (Figures 2, 3, 4, 5) demonstrate the relative strength of each effect in units of scaled mass index. Higher values represent stronger effects of the weather variable being plotted.

Using Akaike's Information Criterion (AIC) we performed model selection using the “dredge” function of the “MuMIn” (Barton, 2009) package in R, which considered all possible models derived from our global models. Instead of using a model averaging approach, we then examined each model with Δ AICc < 2 for biological relevance (Delgado‐Rodríguez & Llorca, 2004; Johnson & Omland, 2004) and selected top models by prioritizing models with two or more of the spline effects of maximum temperature, minimum temperature, and precipitation because they are at the root of our analysis. Finally, we performed a one‐sample t‐test for each species to determine if scaled mass index values were significantly lower in the 2 years following Hurricane Harvey (2018 and 2019) compared to the year preceding Hurricane Harvey (2017) or the 11‐year average from 2007 to 2017. Mean scaled mass index values for each species per year are presented in Table 1.

**TABLE 1:** Mean scaled mass index values for species, as a mean of years 2007–2016, then for the years of 2017, 2018, and 2019 with sample size (*n*) in parenthesis.

Species	Code	Mean (2007–2016)	2017	2018	2019
Northern cardinal	NOCA	41.58 (367)	43.52 (35)	**32.75 (16)**	40.21 (48)
Painted bunting	PABU	15.89 (199)	15.28 (16)	15.33 (13)	15.65 (21)
White‐eyed vireo	WEVI	12.01 (141)	12.29 (29)	11.38 (13)	11.76 (35)

*Note*: Significant values are shown in bold. Hurricane Harvey made landfall in August of 2017, shortly after avian data was collected for that year.

## 3 RESULTS

### 3.1 Model selection

AICc model selection indicated several competing top models with Δ AICc < 2 in each of the three species analyzed: 10 for northern cardinal, 12 for painted bunting, and 26 for white‐eyed vireo. For each species, the models selected for analysis (i.e., our “top models”) ranked higher than the null (intercept only, Table 2). For northern cardinal the Δ AICc of the null model was 40.823, and for painted bunting, 24.717. For white‐eyed vireo, the Δ AICc was 1.892 for the null model. However, our top model for white‐eyed vireo still ranked higher than the null model with a Δ AICc of 0.992. Selected top models were as follows: for northern cardinal, our global model with minimum temperature + maximum temperature + precipitation + age + date + sex + year. For painted bunting, maximum temperature + precipitation + age + date + sex. Lastly, for white‐eyed vireo, minimum temperature + precipitation + date. Our full model selection table for all models with Δ AICc <2 is available in Table S1. Concurvity values for minimum temperature and maximum temperature in our global model exhibited a high level of concurvity (>0.90).

**TABLE 2:** Degrees of freedom (df), AICc, ΔAICc, and model weight (*ω*) for selected models ≤2 ΔAIC for northern cardinal (NOCA), painted bunting (PABU), and white‐eyed vireo (WEVI) with combinations of average daily minimum temperature, average daily maximum temperature, precipitation, age, date, sex, and year covariates. Null models also shown for comparison.

Species	Model	df	AICc	ΔAICc	*ω*
NOCA	MinTemp + MaxTemp + Precip + age + date + sex + year	13	2685.58	1.981	0.04
NOCA	Null	2	2724.43	40.832	1.48e‐10
PABU	MaxTemp + Precip + age + sex + date	11	1061.42	1.617	0.03
PABU	Null	2	1084.52	24.717	2.70e‐07
WEVI	MinTemp + Precip + date	8	661.06	0.996	0.02
WEVI	Null	2	661.96	1.89	0.01

### 3.2 Weather variables

Average daily minimum temperature was included in the top models predicting body condition for northern cardinal and white‐eyed vireo, but not painted bunting. For both species scaled mass index increased with minimum temperature, although for white‐eyed vireo this positive effect only lasted until 13 degrees Celsius, after which scaled mass index declined with minimum temperature (Figure 2). The 95% CIs and 85% CIs both did not overlap with zero for either species, although the lower 95% CI closely approached zero (9.22e‐03) for the white‐eyed vireo (Table 3).

**TABLE 3:** Approximate significance of nonparametric smooth terms of weather covariates.

Species	Parameter	edf	*F*	CI (95%)	CI (85%)	StDev
NOCA	MinTemp	2.16	1.35	1.11e‐02–1.86	2.19e‐02–9.42	0.14
	MaxTemp	2.59	1.89	1.50e‐02–1.98	2.87e‐02–1.04	0.17
	Precip	1.00	0.53	5.36e‐23–8.62e+16	8.60e‐18–5.17e+11	2.15e‐03
PABU	MaxTemp	1.00	3.65	5.44e‐43–8.46 + 35	1.31e‐32–3.52e+25	6.78e‐04
	Precip	4.26	1.75	3.68e‐02–1.20	5.85e‐02–0.76	0.21
WEVI	MinTemp	2.41	1.77	9.22e‐03–0.23	1.42e‐02–0.15	0.05
	Precip	2.26	1.41	1.11e‐02–0.19	1.62e‐02–0.13	0.05

*Note*: Parameters, estimated degrees of freedom (edf), *F*‐value (test for equality to zero for all coefficients that make up a single spline term; Wood, 2013), 95% and 85% confidence intervals, and standard deviation of confidence intervals for the selected model of scaled mass index for each species.

Average daily maximum temperature was a predictor in the top models predicting scaled mass index for northern cardinal and painted bunting. We found a generally negative relationship between average daily maximum temperature and scaled mass index for both species (Figure 3). For northern cardinal, the 95% and 85% CIs did not overlap with zero. However, for painted bunting the lower 95% CI (5.44e‐43) and lower 85% CI (1.31e‐32) both closely approached zero, suggesting a weaker effect (Table 3).

Precipitation predicted scaled mass index for northern cardinal, painted bunting, and white‐eyed vireo, with each species responding to changes in precipitation somewhat differently. For northern cardinal, precipitation, and scaled mass index had a generally positive and linear relationship (Figure 4). However, the lower 95% CI (5.36e‐23) and lower 85% CI (8.60e‐18) closely approached zero, suggesting a weak relationship. Similarly, for white‐eyed vireo we found a positive relationship between precipitation and scaled mass index up to approximately 14 cm, after which a negative relationship was apparent. Painted buntings also exhibited a very similar threshold effect around 14 cm, but more dynamic relationships at very low and high levels of precipitation (Figure 4), which may have resulted from smaller sample sizes at these extremes. The 95% CIs and 85% CIs for both painted bunting and white‐eyed vireo did not overlap with zero (Table 3).

### 3.3 Age and sex

The effects of age and sex were present in the selected models for northern cardinal and painted bunting. However, we only found age to be an important predictor of scaled mass index for northern cardinal (*p* < .001), such that hatch year, or juvenile, individuals had higher scaled mass index scores than adults (Table 4). We found that age was not a significant predictor of body condition in painted buntings, even though it was included in the selected model. Sex (male, female, or unknown) was only present in the selected model for northern cardinal and not for painted bunting or white‐eyed vireo. Female northern cardinals tended to have higher body condition than males (*p* < .001). Neither age, year, nor sex were included in the top model for white‐eyed vireo.

**TABLE 4:** Estimates based on link function, standard error, *t* values, Pr(>|*t*|), and significance for age and year covariates in the selected models for northern cardinal and painted bunting. Neither covariate appeared in the selected model for white‐eyed vireo.

Species	Parameter	Estimate	SE	*t* Value	Pr(>|*t*|)	Significance
NOCA	Age	−2.971	0.876	−3.391	7.63e‐04	***
	Year	−0.104	0.084	−1.237	0.217	
PABU	Age	0.932	0.644	1.447	0.149	

Significance codes: *p* < .001***.

### 3.4 Year

The effect of year was only present in the top model predicting scaled mass index for northern cardinal. Despite this, there was no significant effect of year on scaled mass index (Table 4). However, we also performed a series of one‐sample t‐tests to determine if scaled mass index values were significantly lower in 2018 and 2019, the years following Hurricane Harvey, than in previous years for each species. For northern cardinal, we found that mean scaled mass index in 2018 was significantly lower than that of both 2017 (*t* = −2.15, df = 15, *p* < .05) and the 10‐year average pre‐2018 (*t* = −1.7644, df = 15, *p* < .05). Additionally, we found that average scaled mass index for northern cardinal in 2019, in the second breeding season following Hurricane Harvey, was still significantly lower than that of 2017 (*t* = −7.3954, df = 47, *p* < .001) and of the 11‐year average (*t* = −3.06, df = 47, *p* < .01) (Figure 5). Although we observed a marked increase in average scaled mass index of northern cardinal from 2018 (32.75) to 2019 (40.21), this difference was not statistically significant within the 95% confidence interval (*p* > .05). We did not find any significant differences in the scaled mass index of painted buntings in either 2018 or 2019 compared to the year preceding Hurricane Harvey (2017), or the 11‐year average from 2007 to 2017 (*p* > .05). For white‐eyed vireos, we found that the average scaled mass index in 2019 was notably lower than that in 2017 (*t* = −2.62, df = 34, *p* < .01). However, results from 2018 showed no notable differences (*p* > .05).

## 4 DISCUSSION

Previous literature suggests a complex and dynamic response of avian body condition to weather‐related variables (Gardner et al., 2018), which is consistent with the results of our study. At our long‐term study site on the Texas coast, we found that average daily maximum and minimum temperatures and total precipitation in the prior month were associated with changes in passerine body condition during the breeding season. Our results suggest nonlinear responses to these variables for some species, including threshold effects for both temperature and precipitation, after which associations between weather and body condition reversed. Overall our results indicate that temperature and precipitation can act as important drivers of avian body condition at a local scale, which is also consistent with prior studies focused on nonavian taxa (e.g., springbok; Turner et al., 2012, and kangaroos; Plaisir et al., 2022).

### 4.1 Weather parameters

We found associations between body condition and all weather variables for the species in this study, although the directions of those effects were not easily explained by diet, as we predicted. All species displayed a positive effect of precipitation on body condition at low to moderate levels of precipitation. However, that effect peaked around 14 cm of total precipitation for two of the three species (i.e., white‐eyed vireo and painted bunting), and then reversed. This threshold effect may be due to destruction of cover and food sources because of flooding, and the subsequent displacement of birds (Knutson & Klaas, 1997). Since it is widely established that declines in food availability may lead to declines in avian body condition (McKinnon et al., 2015; Smith et al., 2010), it is possible that the more generalist diet of northern cardinals provided some protection from the potential destructive effects of very high levels of precipitation.

Average daily minimum temperature had a positive effect on scaled mass index of northern cardinal and white‐eyed vireo, although the latter again displayed a threshold effect around 13 degrees Celsius after which minimum temperature had a strongly negative effect on body condition. Increasing minimum temperatures may generally correspond with increased food availability, although very warm minimum temperatures may inhibit insect emergence (Abarca & Spahn, 2021), thus decreasing body condition of the insectivorous species such as the white‐eyed vireo. Alternatively, the smaller‐bodied white‐eyed vireo may be more strongly affected by thermoregulatory requirements at lower minimum temperatures than northern cardinals, necessitating increased fat stores and lower activity levels (Stevenson & Woods, 2006). Average daily maximum temperature was negatively associated with scaled mass index of both northern cardinal and painted bunting, as we predicted, although these effects were weaker for painted bunting. We suspect that this pattern is driven by increasing physiological thermoregulatory stress in individuals as temperatures rise (du Plessis et al., 2012), and/or potential declines in food availability under high heat conditions.

The presence of numerous models with Δ AICc < 2 for northern cardinal, painted bunting, and white‐eyed vireo suggests that the weather variables in our analyses were explaining similar amounts of variation in the dataset, and there was no single weather variable that most strongly explained changes in body condition of these species. For white‐eyed vireo, the null model had a Δ AICc of 1.892, indicating that weather variables only weakly explained variation in scaled mass index for this species and that variation in body condition may be largely driven by nonweather factors or by weather across larger or smaller temporal windows.

### 4.2 Year

Our results indicate that scaled mass index values for northern cardinals in the two breeding seasons following Hurricane Harvey (2018 and 2019) were significantly lower than in previous years (Figure 5). While we were unable to test direct causation due to the correlative nature of this study, a relationship with Hurricane Harvey seems probable due to the strong impact of the storm in this region (Blake & Zelinsky, 2018) and the possibility of the hurricane affecting food resource availability. Other studies have reported higher variance in body condition following high precipitation because of increased nestling survival (Zuckerberg et al., 2018), which could result in lower mean body condition. However, this is unlikely in our case because our precipitation results indicate a generally positive effect of precipitation on body condition for northern cardinals, and Hurricane Harvey probably did not directly affect nestlings because it occurred after the breeding season, although it may have affected fledglings (Lowther et al., 2020). Instead, alteration or destruction of habitat caused by Hurricane Harvey may have negatively impacted food availability (Moreno & Møller, 2011). The sudden, heavy precipitation (i.e., nearly 20 cm in 4 days) associated with this hurricane resulted in extensive flooding on the Welder Wildlife Refuge, which has been previously shown to affect the community composition and species abundance of other avian species (Ferriera de Deus et al., 2020; Royan et al., 2013). Thus, our results suggest that sudden, severe precipitation events may have negative impacts on body condition, despite positive effects of similar precipitation totals over longer periods of time. Year was only present in the top model for northern cardinal, suggesting that our observed potential effects of the hurricane cannot be generalized across all passerine species. The other two species in this study are both migratory species, and thus the impact of a hurricane outside of the breeding season may be felt more strongly on resident than migratory species. This is an avenue of potential future research with a larger, less‐localized data set.

## 5 CONCLUSION

Taken together, our results demonstrate that variation in avian body condition is associated with local temperature and precipitation patterns and that severe weather events may have multiyear impacts on body condition for some species. Body condition is associated with individual fitness for many vertebrate taxa, including birds (Stevenson & Woods, 2006), reptiles (Romero et al., 2001), and mammals (Vervaecke et al., 2005). Reduced body condition can negatively affect foraging success (Jakob et al., 1996) and increase susceptibility to disease and predators (Kouba et al., 2021). Thus, our observed impacts of short‐term weather and an extreme weather event on body condition may have broad implications for individual fitness and population stability (Schoech et al., 2011), although currently, relationships between body condition, climate change, and population dynamics are unclear (see McLean et al., 2018). This is a topic that should be investigated in future studies. Furthermore, since the MAPS program is only run during the primary avian breeding season (May–August), any changes in body condition during other months were not analyzed in this study but could also have important impacts on fitness (Stevenson & Woods, 2006).

Our results also highlight the importance of nonlinear effects of weather, such as threshold effects, for many species. As climate change increases the probability of extreme weather events (Bregman et al., 2016) and continues to impact bird communities (Jenouvrier, 2013), we expect species to be pushed beyond these thresholds more frequently. We also note that these associations between weather thresholds and body condition will likely vary regionally. For example, daily maximum temperatures at our study site between May–August already frequently approach or exceed the point in which northern cardinal body condition declines most steeply in our study (approximately 30 degrees Celsius).

In general, the three species in our study were at greatest risk of low body condition at low minimum temperatures and high maximum temperatures. However, there was large species‐specific heterogeneity in these responses, especially in regard to precipitation. This suggests that responses of avian body condition to weather may not be easily generalized across species, even for passerine species located in the same small geographic area. Future research across a broader spatial scale (e.g., the southeastern United States) may provide further insights as to how local and regional bird populations continue to respond in the years following extreme weather events. Finally, localized studies should also evaluate the effects of weather and reduced body condition on survival and reproductive success.

## AUTHOR CONTRIBUTIONS

**Michael W. D. McCloy:** Conceptualization (equal); data curation (lead); formal analysis (lead); funding acquisition (equal); investigation (equal); methodology (equal); project administration (lead); resources (equal); software (equal); supervision (equal); validation (equal); visualization (lead); writing – original draft (lead); writing – review and editing (lead). **Selma Glasscock:** Conceptualization (equal); data curation (supporting); funding acquisition (equal); investigation (supporting); methodology (supporting); project administration (supporting); resources (equal); supervision (equal); validation (supporting); writing – review and editing (supporting). **Jacquelyn K. Grace:** Conceptualization (equal); data curation (supporting); formal analysis (supporting); funding acquisition (supporting); investigation (supporting); methodology (equal); project administration (equal); resources (supporting); software (supporting); supervision (equal); validation (supporting); visualization (supporting); writing – original draft (supporting); writing – review and editing (equal).

## FUNDING INFORMATION

Funding for this study was made possible through MM's graduate research fellowship with the Rob & Bessie Welder Wildlife Foundation. This manuscript is Welder Contribution Number 737.

## CONFLICT OF INTEREST STATEMENT

The authors declare no conflict of interest.

## Supporting information


Table S1

## Data Availability

The dataset used in this study is freely available via Dryad here: https://doi.org/10.5061/dryad.k6djh9wbc.
