# Supplementary material for: Local weather affects body condition of three North American songbird species on the Texas Coast
Source: Ecol Evol. 2023 Jul 15;13(7):e10317. doi: 10.1002/ece3.10317 (PMC10349279; doi:10.1002/ece3.10317)
Supplement: Supplementary file 1 — Table S1 [file ECE3-13-e10317-s001.docx]

**Supplemental Table 1:** Degrees of freedom (df), AICc, $\Delta$AICc, and model weight ($\omega$) for all models ≤ 2 $\Delta$AIC for northern cardinal (NOCA), painted bunting (PABU), and white-eyed vireo (WEVI) with combinations of average daily minimum temperature, average daily maximum temperature, precipitation, age, date, sex, and year covariates. Selected models shown in bold. Null models also shown for comparison.

| **Species** | **Model** | **df** | **AICc** | $\boldsymbol{\Delta}$**AICc** | $\boldsymbol{\omega}$ |
| --- | --- | --- | --- | --- | --- |
| NOCA | MinTemp + MaxTemp + age + date + sex | 11 | 2683.60 | 0 | 0.11 |
|  | Age + date + sex | 5 | 2684.18 | 0.575 | 0.08 |
|  | MinTemp + MaxTemp + age + date + sex + year | 12 | 2684.30 | 0.700 | 0.07 |
|  | MinTemp + MaxTemp + Precip + age + date + sex | 12 | 2684.68 | 1.083 | 0.06 |
|  | MinTemp + Precip +age + date + sex | 7 | 2684.77 | 1.166 | 0.06 |
|  | MaxTemp + Precip +age + date + sex | 9 | 2684.84 | 1.239 | 0.06 |
|  | Precip + age + date + sex | 6 | 2684.88 | 1.280 | 0.06 |
|  | MaxTemp + age + date + sex | 9 | 2685.43 | 1.419 | 0.05 |
|  | MinTemp + MaxTemp + age + sex + year | 11 | 2685.58 | 1.829 | 0.04 |
|  | **MinTemp + MaxTemp + Precip + age + date + sex + year** | **13** | **2685.67** | **1.981** | **0.04** |
|  | *null* | *2* | *2724.43* | *40.832* | *1.48e-10* |
| PABU | MinTemp + sex | 7 | 1059.80 | 0 | 0.06 |
|  | MinTemp + date + sex | 7 | 1059.80 | 2.84e-04 | 0.06 |
|  | MinTemp + age + sex | 8 | 1059.92 | 0.114 | 0.06 |
|  | MinTemp + age + date + sex | 8 | 1059.92 | 0.116 | 0.06 |
|  | MaxTemp + Precip + sex | 10 | 1060.84 | 1.040 | 0.04 |
|  | MaxTemp + Precip + date + sex | 10 | 1060.84 | 1.040 | 0.04 |
|  | MinTemp + sex + year | 7 | 1060.91 | 1.106 | 0.04 |
|  | MinTemp + date + sex + year | 7 | 1060.91 | 1.106 | 0.04 |
|  | MinTemp + age + sex + year | 8 | 1061.01 | 1.207 | 0.03 |
|  | MinTemp + age + date + sex + year | 8 | 1061.01 | 1.209 | 0.03 |
|  | MaxTemp + Precip + age + sex | 11 | 1061.42 | 1.617 | 0.03 |
|  | **MaxTemp + Precip + age + date + sex** | **11** | **1061.42** | **1.617** | **0.03** |
|  | *null* | *2* | *1084.52* | *24.717* | *2.70e-07* |
| WEVI | MinTemp | 4 | 660.06 | 0 | 0.03 |
|  | Precip | 5 | 660.25 | 0.185 | 0.03 |
|  | Precip + date | 5 | 660.25 | 0.185 | 0.03 |
|  | MinTemp + date | 5 | 660.30 | 0.235 | 0.03 |
|  | MinTemp + year | 5 | 660.59 | 0.526 | 0.02 |
|  | MinTemp + date + year | 5 | 660.59 | 0.527 | 0.02 |
|  | MinTemp + Precip | 7 | 660.87 | 0.804 | 0.02 |
|  | MinTemp + sex | 6 | 661.03 | 0.969 | 0.02 |
|  | **MinTemp + Precip + date** | **8** | **661.06** | **0.996** | **0.02** |
|  | MinTemp + date + sex | 7 | 661.28 | 1.217 | 0.02 |
|  | MaxTemp | 4 | 661.30 | 1.231 | 0.02 |
|  | MaxTemp + date | 4 | 661.30 | 1.234 | 0.02 |
|  | MaxTemp + year | 5 | 661.46 | 1.393 | 0.02 |
|  | MaxTemp + date + year | 5 | 661.46 | 1.393 | 0.02 |
|  | Precip + year | 6 | 661.50 | 1.435 | 0.02 |
|  | Precip + date + year | 6 | 661.50 | 1.435 | 0.02 |
|  | MinTemp + MaxTemp | 5 | 661.74 | 1.672 | 0.01 |
|  | MinTemp + sex + year | 7 | 661.79 | 1.723 | 0.01 |
|  | MinTemp + date + sex + year | 7 | 661.79 | 1.724 | 0.01 |
|  | Precip + sex | 7 | 661.82 | 1.754 | 0.01 |
|  | Precip + date + sex | 7 | 661.82 | 1.754 | 0.01 |
|  | *null* | *2* | *661.96* | *1.892* | *0.01* |
|  | date | 2 | 661.96 | 1.892 | 0.01 |
|  | MaxTemp + Precip | 7 | 661.96 | 1.894 | 0.01 |
|  | MaxTemp + Precip + date | 7 | 661.96 | 1.894 | 0.01 |
|  | MinTemp + MaxTemp + date | 6 | 661.99 | 1.925 | 0.01 |
